# Accumulation of Heavy Metals in Vegetable Species Planted in Contaminated Soils and the Health Risk Assessment

**DOI:** 10.3390/ijerph13030289

**Published:** 2016-03-04

**Authors:** Hang Zhou, Wen-Tao Yang, Xin Zhou, Li Liu, Jiao-Feng Gu, Wen-Lei Wang, Jia-Ling Zou, Tao Tian, Pei-Qin Peng, Bo-Han Liao

**Affiliations:** 1 College of Environmental Science and Engineering, Central South University of Forestry and Technology, Changsha 410004, China; evileyes.z@163.com (H.Z.); trickmen593@126.com (W.-T.Y.); bananaxinxin916@sina.com (X.Z.); liulizxj@163.com (L.L.); gujiaofeng@163.com (J.-F.G.); zoujialing1@126.com (J.-L.Z.); 15700718997@163.com (T.T.); pqpeng123@sina.com (P.-Q.P.); 2 Department of Environmental Science, Changsha Environmental Protection College, Changsha 410004, China; 3 College of Science, Central South University of Forestry and Technology, Changsha 410004, China; wenlei_wang@hotmail.com

**Keywords:** heavy metal, accumulation, health risk, vegetable, target hazard quotient (THQ)

## Abstract

The objectives of the present study were to investigate heavy metal accumulation in 22 vegetable species and to assess the human health risks of vegetable consumption. Six vegetable types were cultivated on farmland contaminated with heavy metals (Pb, Cd, Cu, Zn, and As). The target hazard quotient (THQ) method was used to assess the human health risks posed by heavy metals through vegetable consumption. Clear differences were found in the concentrations of heavy metals in edible parts of the different vegetables. The concentrations of heavy metals decreased in the sequence as leafy vegetables > stalk vegetables/root vegetables/solanaceous vegetables > legume vegetables/melon vegetables. The ability of leafy vegetables to uptake and accumulate heavy metals was the highest, and that of melon vegetables was the lowest. This indicated that the low accumulators (melon vegetables) were suitable for being planted on contaminated soil, while the high accumulators (leafy vegetables) were unsuitable. In Shizhuyuan area, China, the total THQ values of adults and children through consumption of vegetables were 4.12 and 5.41, respectively, suggesting that the residents may be facing health risks due to vegetable consumption, and that children were vulnerable to the adverse effects of heavy metal ingestion.

## 1. Introduction

Heavy metal elements, such as lead (Pb), cadmium (Cd), arsenic (As), *etc.*, have toxic effects on human health. Toxic metals can accumulate persistently in the body over a lifetime. Pb can adversely influence the intelligence development of children, cause excessive lead in blood, and induce hypertension, nephropathy and cardiovascular disease [1,2,3]. Chronic Cd exposure can cause acute toxicity to the liver and lungs, induce nephrotoxicity and osteotoxicity, and impair function of the immune system [4,5,6]. The element As is a metalloid and is associated with angiosarcoma and skin cancer [7,8]. Other metal elements such as copper (Cu) and zinc (Zn) are important nutrients for humans, but excessive ingestion can also have adverse effects on human health [9]. For example, a Cu surplus can cause acute stomach and intestine aches, and liver damage [9,10,11], and Zn can reduce immune function and levels of high-density lipoproteins [11,12]. Compared with inhalation of soil particles, drinking water, and dermal contact, food consumption has been identified as the major pathway for human exposure to toxic metals [13,14].

Vegetables are important edible crops and are an essential part of the human diet. They are rich in nutrients required for human health, and are an important source of carbohydrates, vitamins, minerals, and fibers [15,16]. Heavy metals can be readily taken up by vegetable roots, and can be accumulated at high levels in the edible parts of vegetables, even heavy metal in soil at low levels [16,17]. In many countries and regions, vegetables are exposed to heavy metals by various means, thus vegetable consumption can cause adverse health effects. In Huludao City, China, the ranges of Pb and Cd concentrations in vegetables are 0.003–0.624 mg/kg and 0.003–0.195 mg/kg (fresh weight), respectively, and the maximum concentrations of Pb and Cd all exceed the recommended values (GB 2762-2005) [18]. Hu *et al.* [15] reported that 16%, 26%, and 0.56% of market vegetables in Hong Kong were contaminated by Pb, Cd, and Cr, respectively. Rahman *et al.* [9] reported that some Australian and Bangladeshi vegetables contained Cd concentrations higher than the Australian standard maximum limit (0.1 mg/kg). Therefore, vegetable consumption is considered to be one of the major sources of heavy metal intake for humans, and elevated levels of heavy metal in edible parts of vegetables can affect human health.

Vegetable species differ widely in their ability to take up and accumulate heavy metals, even among cultivars and varieties within the same species [19,20]. Alexander *et al.* [13] reported that Pb significantly accumulated in lettuce and onion, while Cd accumulated to the greatest extents in spinach and lettuce. Yang *et al.* [16] found that Chinese leek, pak choi, and carrot had higher Cd concentrations in their edible parts than radish, cucumber, and tomato. Säumel *et al.* [20] reported that Zn concentrations in green beans, tomato, potato, kahlrabi, and carrots were significantly lower than the concentrations in leafy vegetables. Cd accumulation in vegetable species decreased in the order of leafy vegetables > solanaceous vegetables > root vegetables > allimus vegetables > melon vegetables > legumes vegetables [21].

There are elevated levels of heavy metal in soils in many areas of the world, especially in developing countries and regions. In some mining and smelting areas in China, although the agriculture soils are contaminated with heavy metals, the farmers cannot afford to leave farmland fallow for remediation because the demand and pressure to produce foodstuffs and vegetables are so high. The selection and breeding of crop and vegetable species or cultivars that have low heavy metal accumulation, without having to leave the farmland fallow, seems to be a suitable method to reduce the adverse health effects of heavy metals [21,22,23]. There have been some studies that have focused on Cd contaminated soil and the selection of vegetable species or cultivars with low heavy metal accumulation [16,21,24]. However, very few studies have investigated multiple heavy metals (Pb, Cd, Cu, Zn, and As) in soils. Therefore, in this study, field experiments were carried out on Pb, Cd, Cu, Zn, and As contaminated farmland near Shizhuyuan Mine Zone in Chenzhou City, southern Hunan Province, China, where 22 vegetable species (six vegetable types) were used in the trials. The main purposes of this study were to investigate the concentrations and accumulation of Pb, Cd, Cu, Zn, and As in the edible parts of different vegetable species, and to assess the health risks of vegetable consumption on residents (adults and children).

## 2. Materials and Methods

### 2.1. Experimental Site and Soil

The climate of Shizhuyuan mine area in Suxian District of Chenzhou City is a continental middle subtropical monsoon humid climate zone, with a mean annual temperature in the range 15.6 °C –18.3 °C, a mean annual precipitation in the range 1400–1700 mm, and a frost-free period of about 295 days. The farmland under study (25°52.08′ N, 113°08.98′ E) is located near the East River of Shizhuyuan mine area. In 1985, a tailing dam was burst by a flood due to extreme rainstorms, causing the farmland near the East River to be flooded and covered with toxic tailings [25,26]. The tailings and contaminated top soils were removed by the government immediately after the accident, but there are still some farmlands contaminated with heavy metals that continue to produce foodstuffs and vegetables, which are eaten by the farmers and their families or are taken to the market for sale to urban residents. The farmland soil is contaminated with multiple metals (Pb, Cd, Cu, Zn, and As) in this study, and the basic properties of the farmland soil are given in Table 1.

### 2.2. Field Experiments

A total of 22 vegetable species are in common cultivation in Hunan Province, including three species of root vegetables, two species of stalk vegetables, three species of solanaceous vegetables, four species of melon vegetables, eight species of leafy vegetables, and two species of legume vegetables, and these were cultivated on the experimental farmland in the spring and autumn seasons (Table 2). A rectangular experimental plot area of 8 m^2^ (4 m × 2 m) was established for each vegetable in the farmland. Three replications were conducted for each vegetable. All of the plots were arranged in a randomized complete block design (*n* = 66).

At harvest season (summer and winter, 2011), vegetables were collected at different maturation times. Five vegetable plants from each plot were collected and mixed to obtain a sample. Rhizosphere soil of each vegetable plant was separated from the roots by gently shaking the soil attached to the roots. All samples were stored in polyethylene bags for transport at a constant temperature of 4 °C. The vegetable samples were then cleaned using deionized water to remove dust and soil. The edible parts of the vegetables were separated from the plants, dried in an oven at 105 °C for 30 min, then dried in an oven at 70 °C until the weight of the sample remained constant. All samples were ground to fine powder by using a stainless steel grinder (RT-02B, China), and passed through a 100-mesh sieve, then kept in clean polyethylene container for further analysis. The soils were air-dried at room temperature, ground to fine powder by using an agate mortar and pestle, and passed through a 10-mesh sieve, then kept in clean polyethylene containers before analysis.

### 2.3. Target Hazard Quotient (THQ) Method

The potential health risks of heavy metal consumption through vegetables were assessed based on the target hazard quotient (THQ) method, which was described in detail by the United States Environmental Protection Agency [27,28,29]. The THQ is given by the following equation:

(1) $$\text{THQ}=\frac{E_{F}E_{D}F_{IR}C}{R_{FD}W_{AB}T_{A}}\times 10^{-3}$$

where *E_F_* is the exposure frequency (350 days/year); *E_D_* is the exposure duration (70 years, equivalent to the average lifetime of the Chinese population); *F_IR_* is the food ingestion rate (vegetable consumption values for adults and children are 301.0 and 231.5 g/person/day, respectively) [29]; *C* is the metal concentration in the edible parts of vegetables (mg/kg); *R_FD_* is the oral reference dose (Pb, Cd, Cu, Zn, and As values were 0.0035, 0.001, 0.040, 0.300, and 0.050 mg/kg/day, respectively) [30,31,32]; *W_AB_* is the average body weight (55.9 kg for adults and 32.7 kg for children) [18,29]; and *T_A_* is the average exposure time for non-carcinogens (*E_D_* × 365 days/year). If the THQ value is greater than 1, the exposure is likely to cause obvious adverse effects.

The total THQ (TTHQ) of heavy metals for vegetables is given by the following equation [18,27]:

(2) $$\text{TTHQ}=\sum_{i=1}^{n}(\text{THQ}{)}_{i}$$

### 2.4. Chemical Analysis and Quality Control

Soil pH was measured in 1:2.5 of soil:water suspension using a glass electrode (PHS-3C, LEICI, Guangzhou, China). Soil organic matter (OM) content was determined by oxidation with potassium dichromate and colorimetric determination [33]. Cation exchange capacity (CEC) was determined using the ammonium acetate method after washing with alcohol [34]. The total concentrations of Pb, Cd, Cu, and Zn in soil samples were acid-digested with HCl/HNO_3_/HClO_4_, and the edible parts of the vegetables were digested by means of the dry ashing method [35], and determined using inductively coupled plasma optical emission spectrometry (ICP-OES 6300, Thermo Fisher Scientific, Waltham, MA, USA). The total As concentrations in soil samples were aqua-regia digested, and for the edible parts of the vegetables, a dry ashing method was used [36], and quantification of As by atomic fluorescence spectrometer was conducted (AFS-8220, Titan Instruments, Beijing, China). Certified reference materials for soil (GBW (E) 070009, geochemical certified reference soil of China) and certified reference materials for rice (GBW 10045, certified reference rice of China) were also analyzed with the samples. The standard reference materials were analyzed with the samples during the course of analysis. The mean recoveries of the standard reference materials soil were 95%, 92%, 105%, 92%, and 90% for Pb, Cd, Cu, Zn, and As, respectively, and the mean recoveries of standard reference rice of these metals were 92%, 94%, 98%, 109%, and 88%, respectively.

### 2.5. Statistical Analysis

A one-way ANOVA was used to evaluate the differences among vegetable species. Prior to ANOVA, the homogeneities of the variances were verified using Levene’s test. Duncan’s test was used to detect the significant differences between the means of different vegetable classifications. The criterion for significance in the procedures was set at *p* < 0.05 (significant). All data were presented as arithmetic means with standard error attached. All statistical analyses were conducted using the software Excel 2010 and SPSS Version19.0 (IBM Corporation, New York, NY, USA), and all figures were produced using Origin Version 8.5 software (OriginLab Corporation, Northampton, MA, USA).

## 3. Results

### 3.1. Heavy Metal Concentrations in Vegetable Edible Parts

The concentrations of the heavy metals in the edible parts of the 22 species vegetables are presented in Table 3. As can be seen, clear differences were found in the concentrations of the heavy metals. The observed ranges in the concentrations of Pb, Cd, Cu, Zn, and As in the edible parts were 0.004–2.361 mg/kg, 0.002–2.918 mg/kg, 0.155–3.125 mg/kg, 1.151–54.65 mg/kg, and 0.014–1.780 mg/kg, respectively, with mean concentrations of 0.383, 0.161, 0.810, 10.16, and 0.207 mg/kg, respectively. In all 22 species of vegetables, the Pb concentrations in the edible parts of 15 vegetable species, the Cd concentrations in the edible parts of eight vegetable species, and the As concentrations in the edible parts of four species vegetables were higher than the tolerance limit of contaminants in foods as set by the China National Standards (GB 2762-2012). The lowest concentrations of Pb, Cd, and As in the edible parts of 22 species vegetables were in cucumber (*Cucumis sativus* L.), towel gourd (*Lu*ff*a cylindrica* L.), and tomato (*Lycopersicum esculentum* Mill.), while the highest concentrations were in edible amaranth (*Amaranthus tricolor* L.). In addition, the coefficients of variation of Pb, Cd, Zn, and As concentrations were all greater than 1.0; the coefficient of variation for Cu was 0.85. The difference of Pb, Cd, and As concentrations between the highest and the lowest of the 22 vegetable species reached several hundred times, and even reached a thousand times, while the highest concentrations of Cu and Zn of 22 vegetable species were just a few times the lowest. These results indicated that clear differences in heavy metal (Pb, Cd, Cu, Zn, and As) bioaccumulation existed among the 22 species of vegetables.

### 3.2. Heavy Metal Concentrations in Six Different Vegetable Types

**Figure 1:**
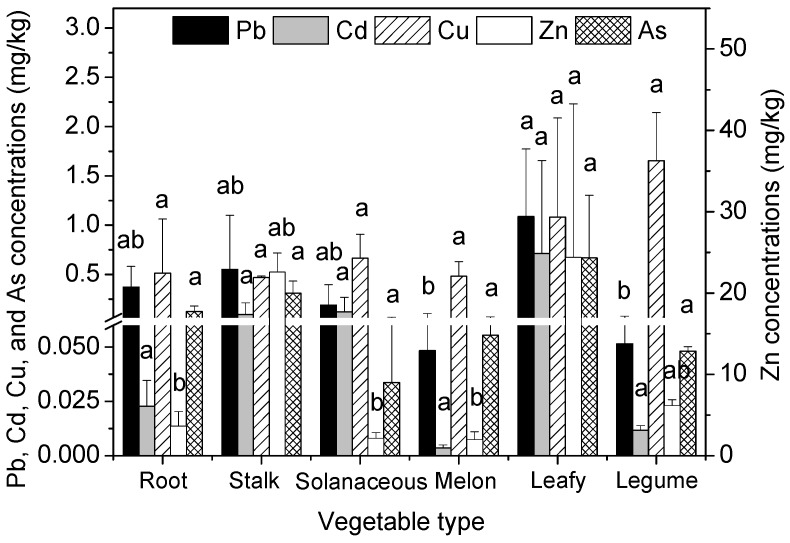
Heavy metal concentrations in the edible parts of six vegetable types (mg/kg, f.w.). Error bars indicate standard error of three replicates. The different letters indicate significant difference at *p* < 0.05 (Duncan’s test).

The heavy metal concentrations in the edible parts of the six different vegetable types are shown in Figure 1. Obvious differences were found in the concentrations of Pb, Cd, Cu, Zn, and As in the edible parts of the six vegetable types. The concentrations in the edible parts of the six vegetable types decreased in the order of leafy vegetables > stalk vegetables > root vegetables > solanaceous vegetables > legume vegetables > melon vegetables for Pb and Cd, legume vegetables > leafy vegetables > solanaceous vegetables > root vegetables > melon vegetables > stalk vegetables for Cu, leafy vegetables > stalk vegetables > legume vegetables > root vegetables > solanaceous vegetables > melon vegetables for Zn, and leafy vegetables > stalk vegetables > root vegetables > melon vegetables > legume vegetables > solanaceous vegetables for As. Consequently, the concentrations of Pb, Cd, Zn, and As in leafy vegetables were significantly higher than the other five vegetable types; however, this was not the case for Cu.

### 3.3. Bioaccumulation Factors (BAFs) of Heavy Metals in Six Vegetable Types

**Figure 2:**
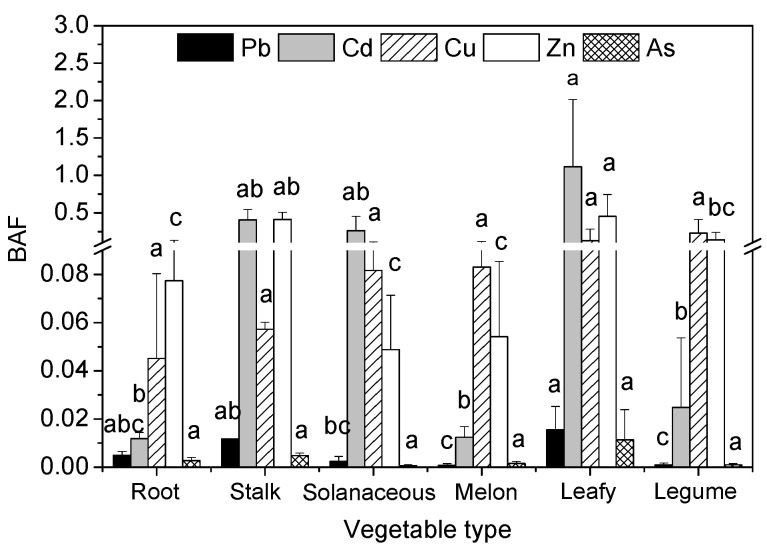
Bioaccumulation factors (BAFs) of heavy metals in six vegetable types. Error bars indicate standard error of three replicates. The different letters indicate significant difference at *p* < 0.05 (Duncan’s test).

Bioaccumulation factors (BAFs), defined as the ratio of the metal concentrations in the edible parts of the vegetable to the metal concentrations in the soil (BAF= C_plant_/C_soil_), can be used to estimate the ability of vegetables to accumulate metals in their edible parts. Significant differences were found in the BAFs of heavy metals in the edible parts of the six vegetable types (Figure 2). The order of the heavy metal (Pb, Cd, Cu, Zn, and As) BAFs was similar to the order of the heavy metal concentrations (Figure 1). The BAFs of Pb, Cd, Cu, Zn, and As in the edible parts of leafy vegetables were higher than for the other five vegetable types. Significant differences (*p* < 0.05) were found in the BAFs of Pb, Cd, and Zn in the edible parts of leafy vegetables and solanaceous vegetables, melon vegetables, and legume vegetables, respectively. The BAFs of all vegetable types were less than 1.0, except for leafy vegetables (1.11). Additionally, the BAFs of Cd, Cu, and Zn were higher than the BAFs of Pb and As (Figure 2). These results indicated that the ability for metal accumulation in the edible parts of leafy vegetables was higher than that for the other five vegetable types, and the ability of Cd, Cu, and Zn accumulation in the edible parts was much greater than that for Pb and As.

### 3.4. Health Risk Assessment of Vegetable Consumption

Chronic low-level intake of toxic metal elements has a negative effect on human health, and the detrimental impact becomes apparent after several years of exposure [30,37,38]. The THQ method was used to assess the potential health risks of heavy metal accumulation through vegetable consumption in this study. Vegetable consumption values for adults and children in China are 301.0 and 231.5 g/person/day, respectively [29]. In the consumption habits of local residents, the leafy vegetable consumption accounted for 65% of total consumption of vegetables, followed by solanaceous and melon vegetables, each of them accounted for 10%, and then each of stalk, root, and legumes vegetables accounted for 5%. The THQ values of Pb, Cd, Cu, Zn, and As due to vegetable consumption for residents (adults and children) of the study area are listed in Table 4. The THQ values of Pb, Cd, Cu, Zn, and As through consumption of leafy vegetables for residents were higher than for the other five vegetable types. For leafy vegetables, the Pb THQ values of adults and children were 1.04 and 1.37, respectively, and Cd THQ values were 2.39 for adults, 3.15 for children, while there were no THQ values for Cu, Zn, and As greater than 1.0 through the consumption of the six vegetable types alone. These results suggested that the potential health risks of heavy metals through consumption of leafy vegetables were the highest for all vegetable types. The THQ values of Pb, Cd, Cu, Zn, and As through vegetable consumption for children were higher than the values for adults in six vegetable types in the present study, indicating that the potential health risks facing for children were greater than for adults. In addition, the total diet THQ of each metal (TDHQ) values of heavy metals decreased in the order Cd > Pb > Zn > Cu > As (Table 4). This suggested that for toxic heavy metals, the potential health risks of Cd and Pb through vegetable consumption were higher than for As.

**Figure 3:**
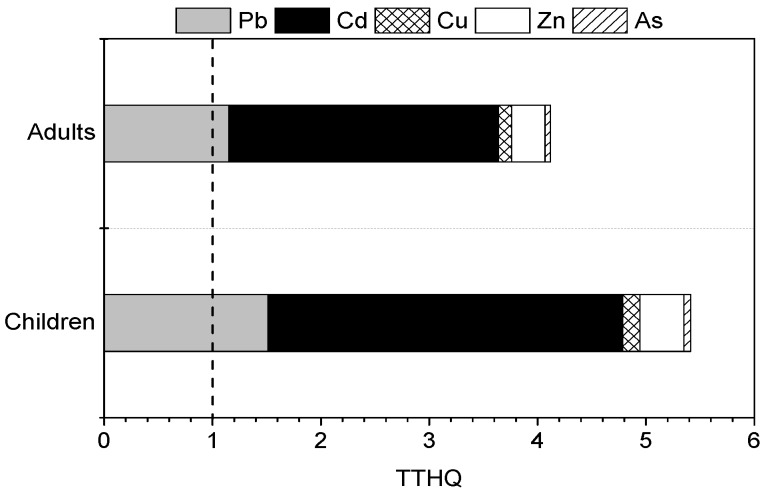
TTHQ values due to consumption of vegetables.

It has been reported that exposure to two or more pollutants may result in additive and/or interactive adverse effects [29]. Therefore, it is hard to assess the potential health risks of multiple metals using each individual THQ value for the heavy metals. Furthermore, the total THQ (TTHQ) of heavy metals is the sum of the individual heavy metal (Pb, Cd, Cu, Zn, and As) THQ values for the six vegetable types, and the values are shown in Figure 3. The TTHQ values for adults and children through vegetable consumption were 4.12 and 5.41, respectively. This result indicated that the residents of the Shizhuyuan area may be facing health risk, and the potential health risks for children were greater than for adults. The relative contributions of Pb, Cd, Cu, Zn, and As to the TTHQ are 27.9%, 60.5%, 3.0%, 7.5%, and 1.2%, respectively. Consequently, Pb and Cd were the main elements contributing to the potential health risks of vegetable consumption for residents in the study area.

## 4. Discussion

Vegetable species differ widely in their ability to take up and accumulate heavy metals, even among cultivars and varieties within the same species [19,20]. It has been reported that Cd uptake and accumulation in leafy vegetables are greater than in non-leafy vegetables [16,21]. In this study, significant differences were found in the concentrations of heavy metals in the edible parts of different vegetable types; the concentrations decreased in the order of leafy vegetables > stalk vegetables/root vegetables/solanaceous vegetables > legume vegetables/melon vegetables (Table 3). In addition, the ability for heavy metal uptake and accumulation of leafy vegetables was higher than for the other vegetable types, and the ability of melon vegetables was the lowest of all vegetable types studied (Figure 2). Edible amaranth, spinach, and caraway had higher concentrations and BAFs of Pb, Cd, Zn, and As (Table 3, Figure 2), and were classed as “high accumulators” [13,19,21]. Lower concentrations and BAFs of Pb, Cd, Zn, and As were found in bitter gourd, towel gourd, cucumber, and pumpkin (Table 3, Figure 2), which were classed as “low accumulators” [13,21]. This suggested that the low accumulators were suitable for being planted on contaminated soil, while the high accumulators were unsuitable [13,19]. The elevated concentration levels of heavy metals and the strong ability for heavy metal accumulation in leafy vegetables were possibly due to the leaves being the main parts of the vegetables used for photosynthesis, because higher metal mass flowed to the leaves due to strong transpiration [39,40,41]; the leaves were also easily exposed to contaminated soil because leafy vegetables were generally dwarfish plants with leaves closer to the ground than the other types of vegetables. Furthermore, atmospheric heavy metal deposition might be one of the reasons for elevated metal concentrations in leafy vegetables in mining and smelting areas [37,42].

Obvious differences in accumulation of heavy metals (Pb, Cd, Cu, Zn, and As) were found in the same vegetable species. The Cd, Cu, and Zn concentrations in soil were lower than Pb and As, while the Cu and Zn concentrations in edible parts were significantly higher than Pb, Cd, and As in all studied vegetables (Table 3). These probably were because Cu and Zn were the essential elements for vegetables growth [9], and were readily accumulated in roots and transported to aerial part [43]; while Pb, Cd, and As were the toxic elements and were not required for vegetables growth, they were stored in roots, and transport to aerial parts of the plant was limited [16,44]. Although Cd concentration in soil was lower than Pb and As concentrations (Table 1), the Cd concentrations in edible parts of vegetables were the same levels as Pb and As (Table 3), and the BAF values of Cd in edible parts were significantly higher than those of Pb and As (Figure 2). These results suggested that Cd had greater accumulation ability in edible parts of vegetables than Pb and As, and the roots were more likely to transport Cd to aerial part [19,43]. Furthermore, the Pb concentrations in edible parts were found to be highly significant positively (*p* < 0.01) correlated with the concentrations of Cd, Zn, and As, and the correlation coefficients (*r*) were 0.872, 0.870, and 0.754 (*n* = 22, *r*_0.01_ = 0.53), respectively; the Cd concentrations were highly significant positively (*p* < 0.01) correlated with the concentrations of Zn and As, and the correlation coefficients (*r*) were 0.806 and 0.784, respectively; the As concentrations were highly significant positively (*p* < 0.01) correlated with the Zn concentrations (*r* = 0.649). It seemed that the absorption, transport, and accumulation of Pb, Cd, As in edible parts might have a relationship with those of Zn.

Generally, Cu and Zn, which are important nutrients for humans, are considered a much lower health risk to humans than Pb, Cd, and As [13]. Poor health can be caused by a lack of these required elements [14], but excessive ingestion can also have adverse effects on human health [9,15]. At present, there are several methods to estimate the potential health risks of pollutants for carcinogenic and non-carcinogenic effects [18,27,29,32]. Non-cancer risk assessment is typically based on the THQ method, which is a ratio of the determined dose of a pollutant to the reference oral dose (*R_FD_*) [27,28,29]. The THQ values are associated with many factors, including intake of pollutants, exposure time, body weight, and reference oral dose of pollutants. The significantly different THQ values for adults and children were due to the differences in the intake of metals, exposure time, and body weight between adults and children in this study (Table 4). Obvious differences had been found in THQ values in males and females through vegetable consumption in Banat Country, Romania, and THQ values for females were higher than those for males [11]. This indicated that the potential health risks for children were higher than those for adults, and that the potential health risks for females were higher than those for males. The TTHQ values of residents in the current study area through vegetable consumption exceeded 1.0 (Figure 3), suggesting that the residents may be facing health risk. Additionally, for special populations, such as those with a weak constitution, those that were sensitive, and women that were pregnant, the potential health risks of heavy metal accumulation through vegetable consumption were likely to be higher than for the normal population. However, vegetable consumption was just one part of food consumption. In addition to vegetable consumption, rice [18,31], meat [18,30,45], fish [29,32], and tobacco [46] consumption also led to ingestion of large amounts of heavy metals. For the residents of Shizhuyuan mining area, food consumption, inhalation of soil particles, drinking water, and dermal contact were the important pathways for human exposure to toxic metals [13,14]. Consequently, the potential health risks for residents were actually higher than the results from this study. Fortunately, the government has realized the adverse effects and the significant health risks posed by heavy metals in recent years, and some remediation measures have already been undertaken on contaminated soils to reduce health risks.

## 5. Conclusions

The present study cultivated 22 species vegetables (six vegetable types) on heavy metal (Pb, Cd, Cu, Zn, and As) contaminated farmland. The concentrations of heavy metals in the edible parts of the vegetables decreased in the order of leafy vegetables > stalk vegetables/root vegetables/solanaceous vegetables > legume vegetables/melon vegetables. The ability for heavy metal uptake and accumulation of leafy vegetables was the highest, and that of melon vegetables was the lowest. These results indicated that the low accumulators (melon vegetables) should be suitable for being planted on contaminated soil, while the high accumulators (leafy vegetables) are unsuitable.

The TTHQ values of adults and children through vegetable consumption were 4.12 and 5.41, respectively, suggesting that the residents of Shizhuyuan area may be facing health risks due to vegetable consumption, and that children were particularly vulnerable to the adverse effects of ingestion of heavy metals. Pb and Cd were the main elements contributing to potential health risks of vegetable consumption for residents in the study area. The potential health risks of heavy metals through other exposure pathways should be the subject of future study.

## Figures and Tables

**Table 1:** Basic properties of the farmland soil under study (d.w.).

Soil Type	pH	CEC (cmol/kg)	Organic Matter (g/kg)	Pb (mg/kg)	Cd (mg/kg)	Cu (mg/kg)	Zn (mg/kg)	As (mg/kg)
Red and yellow soil	6.03	14.62	21.52	1090.0	7.52	128.7	820.0	903.5
Standard for soils ^a^	-	-	-	300	0.3	100	250	30

^a^ Environment quality standard for soils of China (GB 15618-1995).

**Table 2:** Vegetable species used in the experiments.

Vegetable Species	Vegetable Type	Planting Season
White radish (*Raphanus sativus* L.)	Root	Autumn and winter
Carrot (*Daucus carota* L. var. sativa Dc.)	Root	Autumn and winter
Sweet potato (*Ipomoea batatas* (L.) Lam.)	Root	Autumn and winter
White caitai (*Brassica parachinensis* L. H. Bailey in Gent. Herb.)	Stalk	Autumn and winter
Red caitai (*Brassica campestris* L. var. purpurea Baileysh)	Stalk	Autumn and winter
Eggplant (*Solanum melongena* L.)	Solanaceous	Spring and summer
Red pepper (*Capsicum annuum* Linn.)	Solanaceous	Spring and summer
Tomato (*Lycopersicum esculentum* Mill.)	Solanaceous	Spring and summer
Bitter gourd (*Momordica charantia* Linn.)	Melon	Spring and summer
Towel gourd (*Lu*ff*a cylindrica* L.)	Melon	Spring and summer
Cucumber (*Cucumis sativus* L.)	Melon	Spring and summer
Pumpkin (*Cucurbita moschata* Duch.)	Melon	Spring and summer
Mater convolvulus (*Ipomoea aquatica* Forsk.)	Leafy	Spring and summer
Edible amaranth (*Amaranthus tricolor* L.)	Leafy	Spring and summer
Cabbage (*Brassica oleracea* L. var. capitata Linn.)	Leafy	Autumn and winter
Pai-tsai (*Brassica chinensis* Linn.)	Leafy	Autumn and winter
Chinese cabbage (*Brassica pekinensis* (*Lour.*) *Rupr.*)	Leafy	Autumn and winter
Spinach (*Spinacia oleracea* L.)	Leafy	Autumn and winter
Caraway (*Coriandrum sativum* L.)	Leafy	Autumn and winter
Lettuce (*Lactuca satiua* L.)	Leafy	Autumn and winter
Asparagus bean (*Vigna unguiculata* subsp. *sesquipedalis* L.)	Legume	Spring and summer
Kidney bean (*Phaseolus vulgaris* L.)	Legume	Spring and summer

**Table 3:** Concentrations of heavy metals in vegetable edible parts (mg/kg, f.w.). Data show mean ± standard error of three replicates.

Vegetable Type	Vegetable Species	Pb (mg/kg)	Cd (mg/kg)	Cu (mg/kg)	Zn (mg/kg)	As (mg/kg)
Root (*n* = 3)	White radish	0.270 ± 0.057	0.011 ± 0.003	0.167 ± 0.073	4.690 ± 1.367	0.099 ± 0.012
	Carrot	0.233 ± 0.001	0.023 ± 0.005	0.227 ± 0.011	1.591 ± 0.092	0.188 ± 0.030
	Sweet potato	0.613 ± 0.162	0.135 ± 0.007	0.015 ± 0.005	4.674 ± 0.790	0.448 ± 0.013
	National standard ^a^	0.1	0.1	/	/	0.5
Stalk (*n* = 2)	White caitai	0.785 ± 0.239	0.239 ± 0.090	0.456 ± 0.033	24.23 ± 5.541	0.225 ± 0.043
	Red caitai	0.939 ± 0.327	0.176 ± 0.038	0.478 ± 0.089	20.95 ± 2.993	0.396 ± 0.076
	National standard ^a^	0.3	0.1	/	/	0.5
Solanaceous (*n* = 3)	Eggplant	0.429 ± 0.001	0.289 ± 0.027	0.937 ± 0.199	2.786 ± 0.588	0.072 ± 0.004
	Red pepper	0.056 ± 0.009	0.047 ± 0.035	0.589 ± 0.124	2.241 ± 0.169	0.016 ± 0.005
	Tomato	0.078 ± 0.023	0.028 ± 0.005	0.468 ± 0.036	1.419 ± 0.185	0.014 ± 0.003
	National standard ^a^	0.1	0.05	/	/	0.5
Melon (*n* = 4)	Bitter gourd	0.061 ± 0.010	0.002 ± 0.001	0.505 ± 0.127	2.769 ± 0.710	0.061 ± 0.009
	Towel gourd	0.007 ± 0.000	0.002 ± 0.001	0.487 ± 0.030	1.151 ± 0.111	0.048 ± 0.002
	Cucumber	0.004 ± 0.001	0.004 ± 0.001	0.284 ± 0.047	1.206 ± 0.107	0.039 ± 0.014
	Pumpkin	0.121 ± 0.031	0.005 ± 0.001	0.647 ± 0.104	2.883 ± 0.749	0.073 ± 0.015
	National standard ^a^	0.1	0.05	/	/	0.5
Leafy (*n* = 8)	Mater convolvulus	0.510 ± 0.066	0.203 ± 0.099	3.125 ± 0.903	7.055 ± 0.303	1.440 ± 0.257
	Edible amaranth	2.361 ± 0.287	2.918 ± 0.592	1.981 ± 0.103	54.65 ± 12.58	1.780 ± 0.300
	Cabbage	0.671 ± 0.277	0.036 ± 0.022	0.314 ± 0.043	9.926 ± 2.226	0.211 ± 0.014
	Pai-tsai	0.418 ± 0.062	0.121 ± 0.017	0.345 ± 0.011	23.66 ± 4.135	0.125 ± 0.028
	Chinese cabbage	0.749 ± 0.129	0.419 ± 0.027	0.155 ± 0.024	14.51 ± 2.811	0.073 ± 0.020
	Spinach	0.971 ± 0.194	0.513 ± 0.055	0.966 ± 0.288	20.81 ± 4.543	0.310 ± 0.064
	Caraway	1.855 ± 0.208	1.031 ± 0.064	0.987 ± 0.037	52.69 ± 6.200	0.739 ± 0.075
	Lettuce	1.162 ± 0.540	0.460 ± 0.207	0.775 ± 0.170	11.79 ± 1.688	0.660 ± 0.086
	National standard ^a^	0.3	0.2	/	/	0.5
Legume (*n* = 2)	Asparagus bean	0.070 ± 0.014	0.013 ± 0.011	1.999 ± 1.247	6.682 ± 1.550	0.047 ± 0.014
	Kidney bean	0.033 ± 0.036	0.010 ± 0.017	1.310 ± 0.085	5.669 ± 0.236	0.050 ± 0.010
	National standard ^a^	0.2	0.1	/	/	0.5
Range		0.004–2.361	0.002–2.918	0.155–3.125	1.151–54.65	0.014–1.780
Arithmetic mean		0.383	0.161	0.810	10.159	0.207
Standard deviation		0.396	0.275	0.474	10.448	0.248
Coefficient of variation		1.034	1.706	0.585	1.029	1.199

^a^ The tolerance limit of contaminants in foods in the China National Standards (GB 2762-2012).

**Table 4:** Heavy metal target hazard quotient (THQ) values due to consumption of six vegetable types.

Vegetable Type	Adults					Children				
	Pb	Cd	Cu	Zn	As	Pb	Cd	Cu	Zn	As
Root	0.027	0.006	0.003	0.003	0.001	0.036	0.008	0.004	0.004	0.001
Stalk	0.041	0.024	0.003	0.019	0.002	0.053	0.031	0.004	0.026	0.002
Solanaceous	0.028	0.063	0.009	0.004	0.0003	0.036	0.083	0.011	0.005	0.001
Melon	0.007	0.002	0.006	0.003	0.001	0.009	0.002	0.008	0.005	0.001
Leafy	1.04	2.39	0.091	0.273	0.045	1.37	3.15	0.119	0.359	0.059
Legume	0.004	0.003	0.011	0.005	0.0002	0.005	0.0040	0.014	0.007	0.0003
TDHQ ^a^	1.15	2.49	0.123	0.308	0.048	1.51	3.27	0.161	0.405	0.063

^a^ The total diet THQ of each metal (total diet, *i.e.*, the sum of root, stalk, solanaceous, melon, leafy, and legume).
